# A Staggered Vane-Shaped Slot-Line Slow-Wave Structure for W-Band Dual-Sheet Electron-Beam-Traveling Wave Tubes

**DOI:** 10.3390/s24123709

**Published:** 2024-06-07

**Authors:** Yuxin Wang, Jingyu Guo, Yang Dong, Duo Xu, Yuan Zheng, Zhigang Lu, Zhanliang Wang, Shaomeng Wang

**Affiliations:** 1 School of Electronic Science and Engineering, University of Electronic Science and Technology of China, No. 2006 Xiyuan Avenue, High-Tech District (West District), Chengdu 611731, China; 202211022437@std.uestc.edu.cn (Y.W.); 202111022436@std.uestc.edu.cn (J.G.); 202111022405@std.uestc.edu.cn (Y.D.); d.xu@uestc.edu.cn (D.X.); zyzheng@uestc.edu.cn (Y.Z.); lzhgchnn@uestc.edu.cn (Z.L.); wangzl@uestc.edu.cn (Z.W.); 2 Yangtz Delta Region Institute (Quzhou), University of Electronic Science and Technology of China, No. 1, Chengdian Road, Quzhou 324003, China

**Keywords:** slot line, slow-wave structure (SWS), W-band, dual sheet electron beam, traveling wave tube

## Abstract

A staggered vane-shaped slot-line slow-wave structure (SV-SL SWS) for application in W-band traveling wave tubes (TWTs) is proposed in this article. In contrast to the conventional slot-line SWSs with dielectric substrates, the proposed SWS consists only of a thin metal sheet inscribed with periodic grooves and two half-metal enclosures, which means it can be easily manufactured and assembled and has the potential for mass production. This SWS not only solves the problem of the dielectric loading effect but also improves the heat dissipation capability of such structures. Meanwhile, the SWS design presented here covers a −15 dB *S*_11_ frequency range from 87.5 to 95 GHz. The 3-D simulation for a TWT based on the suggested SWS is also investigated. Under dual-electron injection conditions with a total voltage of 17.2 kV and a total current of 0.3 A, the maximum output power at 90 GHz is 200 W, with a 3 dB bandwidth up to 4 GHz. With a good potential for fabrication using microfabrication techniques, this structure can be a good candidate for millimeter-wave TWT applications.

## 1. Introduction

Miniaturized vacuum electronics are commonly employed in millimeter-wave bands for applications such as high-data-rate wireless communications, satellite communications, and high-resolution radars [1,2]. Among them, the planar traveling wave tube (TWT) [3,4,5] is one of the most promising vacuum electronic devices due to its simple structure and compatibility with microfabrication methods in the millimeter-wave band.

One core part of a TWT is its slow-wave structure (SWS), which serves to slow down and synchronize the electromagnetic waves with a high-energy electron beam. Meander-line SWSs [6,7,8,9] have been extensively utilized in planar SWSs due to their low operating voltage and mass manufacturing capability. However, meander-line (ML) SWSs are typically kept in place by dielectric rods or dielectric substrates, which are susceptible to charge accumulation effects that can cause destructive damage to the device [10,11].

Dielectric slab-supported slot-line SWS [12] and co-planar SWS [13] can effectively minimize the dielectric’s exposed area, thereby reducing the probability of charges bombarding the dielectric. The backward wave has a higher coupling impedance; hence, this SWS is commonly employed in backward wave oscillators (BWOs). However, the presence of the dielectric limits the thickness of the metal layer in these two types of SWSs, and in actual processing, the loss of the intermediate seed layer, which connects the dielectric to the metal layer, can have a negative impact on wave transmission [14].

Due to the size co-transition effect, the transverse dimension of the SWS decreases with increasing frequency, and then the use of large compression ratios for multiple sheet electron beam injections can effectively increase the output power. Several designs of multiple-tunnel TWTs operating in millimeter-wave bands have been proposed [15,16].

In this condition, a novel planar-staggered vane-shaped slot-line (SV-SL) SWS is proposed. This slot-line SWS dispenses with a dielectric substrate and consists of a metal sheet and a metal shell. The structure operates in the traveling wave region by adjusting the structural parameters. Similar to the meander-line SWS, the SV-SL SWS is simple to fabricate, can be mass-produced, and has better heat dissipation characteristics. Meanwhile, this SWS has two natural electron injection channels, which can be used for dual-electron injection operations and can effectively increase the output power.

## 2. SWS Design and Discussion

### 2.1. Design and Electromagnetic Parameters

**Figure 1:**
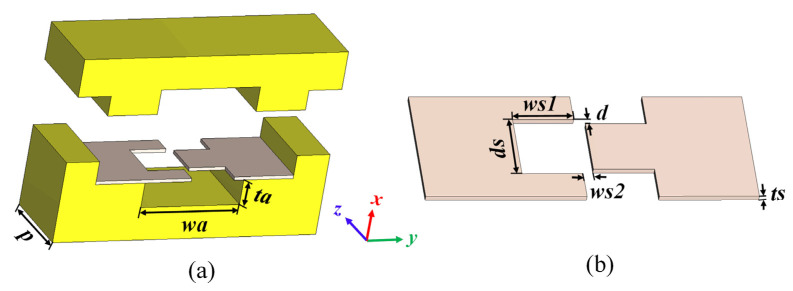
Schematic of the single period SV-SL SWS. (**a**) Perspective view of the proposed SWS with cut shell and (**b**) cross-section view of staggered vane slot line.

The schematic design of the proposed SV-SL SWS with natural dual beam tunnels is shown in Figure 1. This model is partitioned into the following three parts: the upper shell, the middle sheet, and the lower shell. The distance between the upper and lower shells is drawn out for easy observation. The center part is engraved with staggered vane patterns and fixed between the grooved upper and lower shells, while the gaps between the shell and centerpiece become nature beam tunnels. The dimensions of the structure are presented in Table 1.

**Figure 2:**
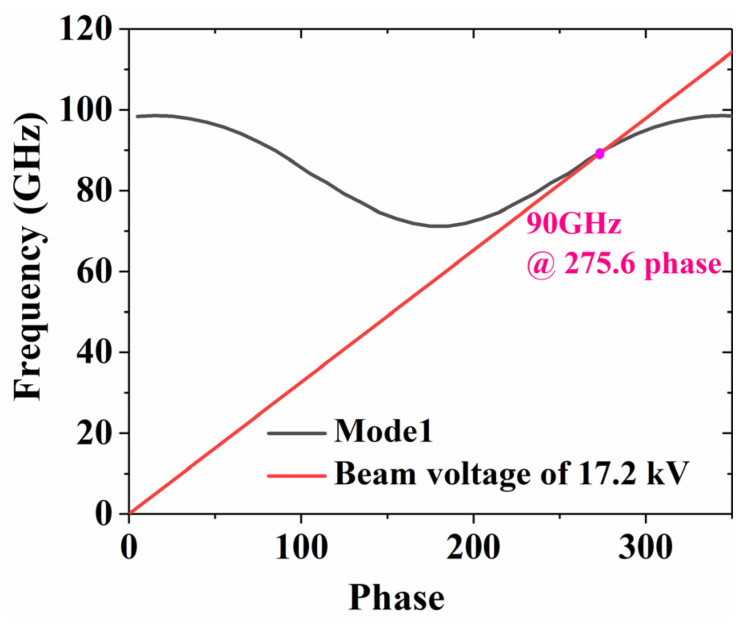
The dispersion diagram of the proposed SWS.

We simulated a single period of the proposed SWS with Floque periodic boundary conditions along the axial direction. The results are presented in Figure 2. To effectively avoid the occurrence of backward wave oscillations and band-edge oscillations, the electron injection voltage line should avoid intersecting the backward wave region (with phase shifts ranging from 0° to 180°) and move away from points with phase shifts of π and 2π. Therefore, by increasing the length of the single period and decreasing the height of the electron injection tunnels, the 17.2 kV beam line and the fundamental mode of the SWS intersect in the forward wave region at 90 GHz, which means that signals near this frequency may be amplified.

**Figure 3:**
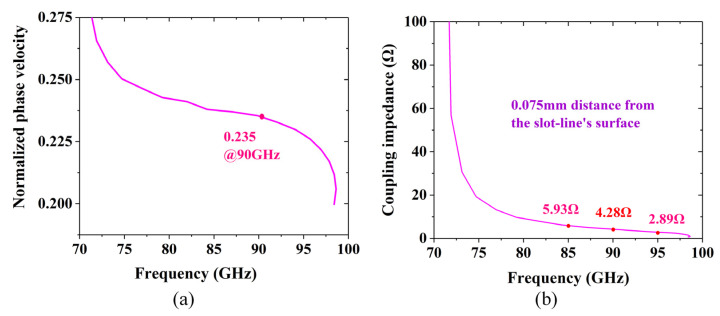
(**a**) Normalized phase velocity curve and (**b**) coupling impedance curve of the SV-SL SWS with frequency.

In addition, the variation in the normalized phase velocity and coupling impedance with frequency is shown in Figure 3. In the frequency range of 85–95 GHz, the dispersion characteristic curves are relatively flat, and the coupling impedance calculated for the fundamental forward harmonic mode of the SV-SL SWS is about 2.89–5.93 Ω at 0.075 mm from the surface of the slot line.

### 2.2. S-Parameters

**Figure 4:**
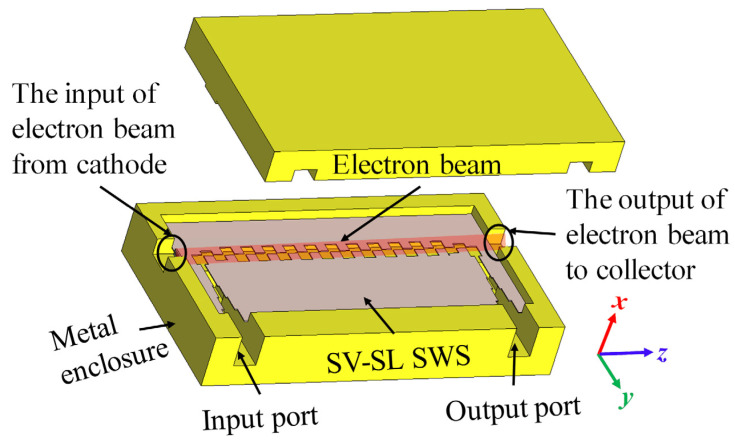
Assembly model of the proposed SWS with coupling structures.

Figure 4 depicts the whole assembly model of the proposed SWS with coupling devices. A thin central sheet of metal is laser-engraved with periodic staggered vane grooves, and the metal enclosure is divided into two halves that can be fabricated with a computer numerical control (CNC) milling or casting machine. The gap between the middle metal sheet and the upper and lower enclosures can serve as a dual electron injection channel.

**Figure 5:**
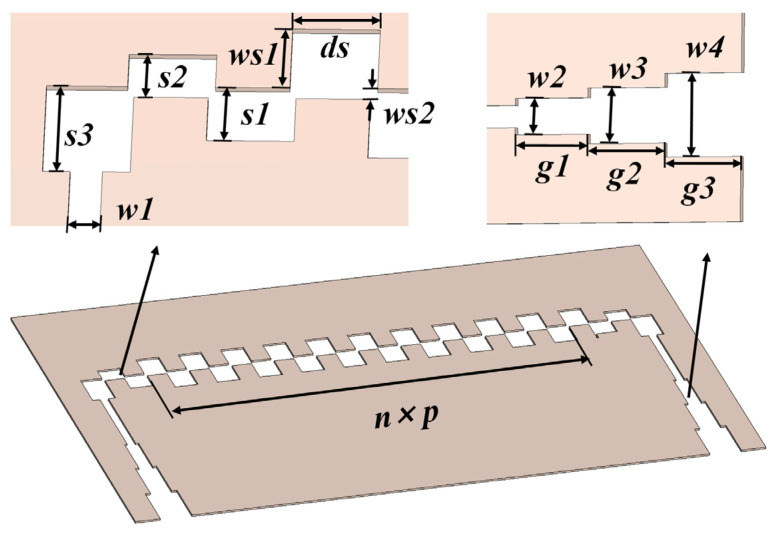
Schematic of the SV-SL SWS with transition coupler parts.

To eliminate extra reflections, a transition section, as seen in Figure 5, is employed to connect the SWS to the input/output structure. A stepped ridge waveguide is usually adopted as the transmission transition structure for a planar meander-line SWS, where impedance matching can be achieved by adjusting the height of the ridge waveguide (the dimension along the x-direction); during this process, the transition from the TE_10_ mode of the standard rectangular waveguide to the quasi-TEM mode of the meander line can be realized. However, this matching method requires the meander-line and ridge waveguide to be processed separately and then assembled as a whole unit, which is troublesome to accurately assemble and prone to introduce more assembly errors. In order to simplify the assembly of the whole SWS, in this SV-SL SWS, we controlled the height of the ridge waveguide in the x-direction to match the thickness of the centerpiece, i.e., the beam tunnel height, and achieved impedance matching by adjusting the dimensions of the ridge waveguide in the z- and y-directions. The dimensional parameters are listed in Table 2.

**Figure 6:**
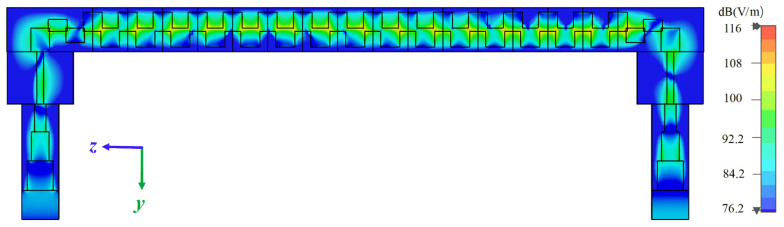
Electric field distribution of the transmission model of the proposed SWS.

Meanwhile, the electric field distribution of the transmission model of the proposed SWS is shown in Figure 6. After optimizing the dimensional parameters of the connection portion, this model can complete the wave transmission.

To examine the transmission characteristics of the SWS, we simulated a 50-period circuit with input/output couplers on both sides using the CST Studio Suite simulator [17].

In our previous research [18], the effects of laser cutting, acid cleaning, and copper plating on the surface roughness and S-parameter of the meander slot-line were investigated. The experimental results show that after plating a 3–5 µm copper layer on the surface of the laser-cut molybdenum sheet, the surface roughness reaches 1.67 μm, and its effective conductivity is 1.48 × 10^7^ S/m. Therefore, in the SV-SL SWS simulation, the upper and lower shells are assumed to be copper with a conductivity of 3 × 10^7^ S/m. And the central slot-line is considered to be a molybdenum sheet with an effective conductivity of 1.48 × 10^7^ S/m; the effective conductivity *σ_ef_* was calculated according to the well-known formula.

(1) $$\delta =\sqrt{\frac{2}{\omega \mu \sigma }}$$

(2) $$\sigma_{ef}=\frac{\sigma }{\left(1+\frac{2}{\pi }\arctan(1.4\times {\left(\frac{R}{\delta }\right)}^{2})\right)}$$

where the *δ* is the skin depth, ω is the angular frequency, *μ* = 4π × 10^−7^ H/m is the magnetic conductivity, *σ* = 5.8 × 10^7^ S/m is the bulk conductivity of copper, and *R* is the surface roughness.

**Figure 7:**
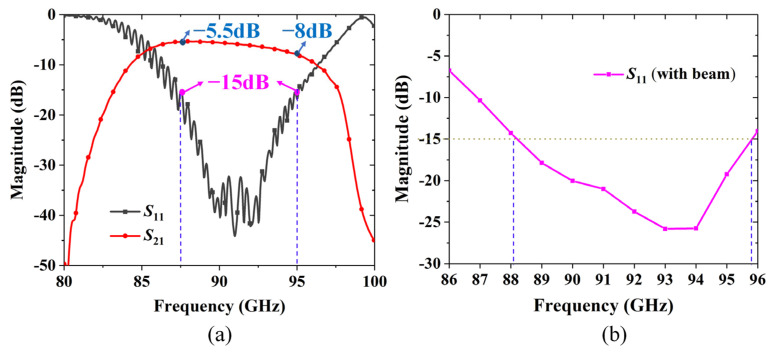
(**a**) Transmission characteristics of the 50-period SWS with input/output couplers. (**b**) *S*_11_ at the interaction of the wave with an electron beam in the manuscript.

The obtained S-parameters are presented in Figure 7a. In the frequency range of 87.5–95 GHz, the reflection loss *S*_11_ does not exceed −15 dB (black line), and the transmission loss *S*_21_ varies from −5.5 dB to −8 dB (red line). Due to the influence of high-frequency loss, the value of *S*_21_ decreases significantly with frequency (the value of *S*_21_ is negative while the absolute value of *S*_21_ increases). During the actual processing, the meander line SWS with the dielectric support or substrate needs to consider the loss of the intermediate seed layer connecting the dielectric layer and the metal layer, which causes a serious negative impact on wave transmission [19,20]. Clearly, the transmission loss of SV-SL SWS with the seed layer removed is much lower.

The *S*_11_ at the interaction of the wave with the electron beam is calculated through the input and reflected power at different frequencies, and the simulation results are shown in Figure 7b, which are less than −15 dB within the frequency 88–95 GHz and are in good agreement with the results in Figure 7a.

## 3. Beam-Wave Interaction Simulation

To evaluate the output performance of the W-band TWT with the proposed dual-beam SV-SL SWS, we used the 3-D particle-in-cell (PIC) CST Particle Studio simulator [21]. In the simulation, we considered two identical 0.15 A electron beams (with a total current of 0.3 A) focused by a uniform magnetic field. And the beam voltage was 17.2 kV. The axis of the electron beam was positioned at 0.075 mm above the SWS. Considering the electron beam tunnel size, the transversal dimension of the beams was 0.8 mm × 0.1 mm (current density of 187.5 A/cm^2^), corresponding to a broadside filling factor of 40%. The total length of the SWS with 50 periods plus the input/output coupling section was 66 mm. Considering the stable beam transportation without current interception by the beam tunnel walls, the uniform magnetic field should be 0.6 T or higher.

**Figure 8:**
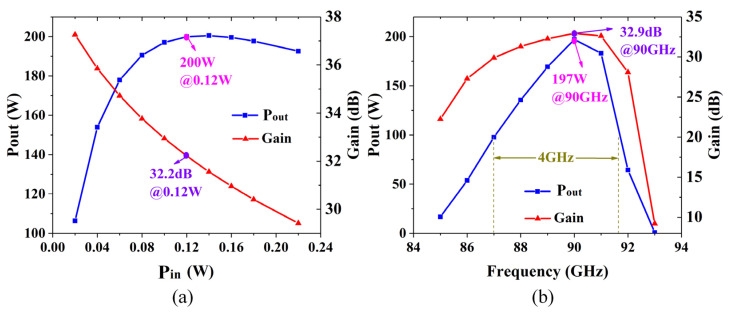
(**a**) Output power versus the input power at 90 GHz. (**b**) Output power versus frequency with 0.1 W input power.

Figure 8a depicts the output power and gain versus input power plot. The simulation maximum output power can reach 200 W with an input power of 0.12 W, and the corresponding gain is 32.2 dB at 90 GHz. By contrast, in Figure 8b, the driving power is set to 0.1 W. The simulation forecasts a maximal gain of 32.9 dB at 90 GHz, and the corresponding output power of 197 W. And the 3 dB bandwidth is 4 GHz.

**Figure 9:**
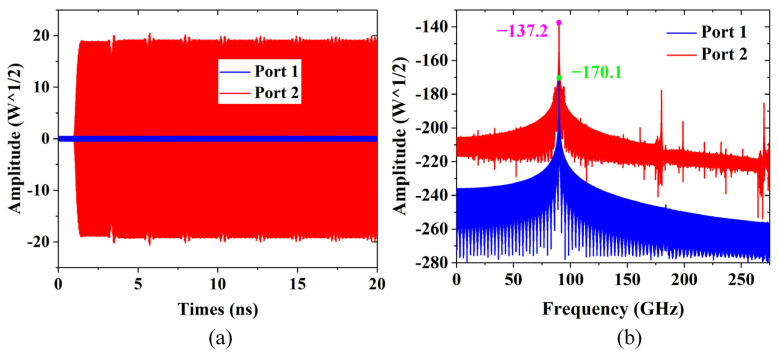
(**a**) Input and output signals versus time for SWS at 17.2 kV beam voltage. (**b**) The spectrum of the input and output signals.

As shown in Figure 9a, we simulated the fluctuation of the SWS output signal with time at a 17.2 kV beam voltage, 90 GHz frequency, and 0.1 W input power. The signal is input from port 1, and after wave injection interaction, it is output from port 2, indicating that there is no noticeable oscillation and that it can be amplified stably. The output signal is stable across the whole frequency range, and we observed a clean single-frequency spectrum with no spurious mode excitation, as illustrated in Figure 9b.

**Figure 10:**
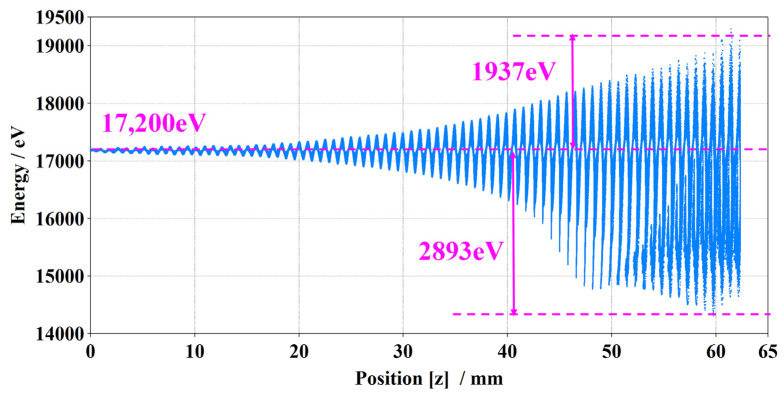
Electron beam phase-space diagram.

Figure 10 shows the corresponding beam phase-space diagram. It indicates the effective beam bunching along the axial direction. Most particles lose energy, and a few electrons gain energy, showing a sufficient beam wave energy transfer.

According to studies [20,22,23,24,25,26], as shown in Table 3, the saturated output power of microstrip-like structures is generally limited by the small size of the structure, which leads to low operating voltages and currents. Moreover, microstrip-like structures are usually held in place by dielectric substrates, which are susceptible to charge accumulation effects, and the meander line SWS using a dielectric substrate needs to take into account the high loss of the intermediate seed layer connecting the dielectric layer to the metal layer in actual processing, so *S*_21_ in experiments is usually below −30 dB. The SV-SL SWS has a higher operating voltage, and its saturated output power can be more than twice that of the microstrip-like structure at approximately the same 3 dB bandwidth. Since the SV-SL SWS removes the seed and dielectric layers and adopts an all-metal structure, it is easier to process and assemble, and the transmission loss is smaller. Subsequent work should ensure that the operating voltage is reduced as much as possible and the efficiency of the electron interaction is improved with little change in bandwidth.

## 4. Conclusions

In conclusion, we proposed a dual-beam SV-SL SWS and investigated its application in W-band TWT. The SWS was found to have suitable coupling impedance and gentle dispersion characteristics. The simulated transmission characteristics of the 50-pitch structure with the input/output couplers demonstrated good performance in the 87.5–95 GHz frequency range. The transmission loss was substantially lower than the meander-line SWS with dielectric support or substrate. According to the 3-D PIC simulation, when set to a dual beam with 17.2 kV and 0.3 A in total, the maximum output power can reach 200 W at 90 GHz, corresponding to the maximum gain and efficiency of 32.2 dB and 3.63%, respectively. The all-metal SV-SL SWS consists of the following three parts: the metal sheet in the middle is carved by a laser engraving or wire cutting to form a periodic winding groove; the upper and lower metal shells are manufactured by a CNC milling machine or casting; and they are then fixed by resistance welding or brazing. Compared with the microstrip-like structures, SV-SL SWS not only completely solves the charge accumulation problem caused by the dielectric substrate and the loss caused by the seed layer, but it also has good mechanical properties that can account for both thermal properties and large-scale production. As a result, the suggested SV-SL SWS has significant application potential in the W-band TWT.

## Figures and Tables

**Table 1:** Dimension parameters.

Parameters	Dimension (mm)	Parameters	Dimension (mm)
*p*	1.2	*ws*1	0.55
*wa*	1.5	*ws*2	0.1
*ta*	0.375	*d*	0.05
*ds*	0.65	*ts*	0.05

**Table 2:** Dimension parameters for transition.

Parameters	Dimension (mm)	Parameters	Dimension (mm)
*s*1	0.5	*w*3	0.6
*s*2	0.4	*w*4	0.9
*s*3	0.8	*g*1	0.95
*w*1	0.24	*g*2	1
*w*2	0.39	*g*3	1

**Table 3:** Comparison of different planar SWSs in the W-band.

Type	Operating Parameters	Simulation Output Power, Efficiency and BW
Metalized ML TWT [20]	9 kV, 0.028 A	10 W, 4%, 3 GHz
ML SWS with the CVD diamond substrate [22]	15.6 kV, 0.043 A	40 W, 5.96%, 5 GHz
PML SWS (cylindrical beam) [23]	6.5 kV, 0.04 A	36 W, 1.38%, 5 GHz
U-shaped ML SWS [24]	7.1 kV, 0.1 A	20.77 W, 2.9%, 7 GHz
ML SWS with a conformal substrate [25]	6.55 kV, 0.1 A	31.4 W,4.8%, 6 GHz
Miniature ML SWS [26]	14 kV, 0.2 A	121 W, 4.32%, 3.5 GHz
SV-SL SWS	17.2 kV, 0.3 A	200 W, 3.63%, 4 GHz

## Data Availability

No new data were created or analyzed in this study. Data sharing is not applicable to this article.
